# Genome-wide repeat dynamics reflect phylogenetic distance in closely related allotetraploid *Nicotiana* (Solanaceae)

**DOI:** 10.1007/s00606-016-1356-9

**Published:** 2016-11-01

**Authors:** Steven Dodsworth, Tae-Soo Jang, Monika Struebig, Mark W. Chase, Hanna Weiss-Schneeweiss, Andrew R. Leitch

**Affiliations:** 1 School of Biological and Chemical Sciences, Queen Mary University of London, London, E1 4NS UK; 2 Department of Comparative Plant and Fungal Biology, Royal Botanic Gardens, Kew, Richmond, Surrey TW9 3DS UK; 3 Department of Systematic and Evolutionary Botany, University of Vienna, Rennweg 14, 1030 Vienna, Austria; 4 School of Plant Biology, University of Western Australia, Crawley, WA 6009 Australia

**Keywords:** Chromoviruses, Graph-based clustering, High-throughput sequencing, Phylogenetics, Repetitive DNA, Ty-3 Gypsy

## Abstract

*Nicotiana* sect. *Repandae* is a group of four allotetraploid species originating from a single allopolyploidisation event approximately 5 million years ago. Previous phylogenetic analyses support the hypothesis of *N. nudicaulis* as sister to the other three species. This is concordant with changes in genome size, separating those with genome downsizing (*N. nudicaulis*) from those with genome upsizing (*N. repanda*, *N. nesophila*, *N. stocktonii*). However, a recent analysis reflecting genome dynamics of different transposable element families reconstructed greater similarity between *N. nudicaulis* and the Revillagigedo Island taxa (*N. nesophila* and *N. stocktonii*), thereby placing *N. repanda* as sister to the rest of the group. This could reflect a different phylogenetic hypothesis or the unique evolutionary history of these particular elements. Here we re-examine relationships in this group and investigate genome-wide patterns in repetitive DNA, utilising high-throughput sequencing and a genome skimming approach. Repetitive DNA clusters provide support for *N. nudicaulis* as sister to the rest of the section, with *N. repanda* sister to the two Revillagigedo Island species. Clade-specific patterns in the occurrence and abundance of particular repeats confirm the original (*N. nudicaulis* (*N. repanda* (*N. nesophila* + *N. stocktonii*))) hypothesis. Furthermore, overall repeat dynamics in the island species *N. nesophila* and *N. stocktonii* confirm their similarity to *N. repanda* and the distinctive patterns between these three species and *N. nudicaulis*. Together these results suggest that broad-scale repeat dynamics do in fact reflect evolutionary history and could be predicted based on phylogenetic distance.

**Electronic supplementary material:**

The online version of this article (doi:10.1007/s00606-016-1356-9) contains supplementary material, which is available to authorized users.

## Introduction

**Fig. 1:**
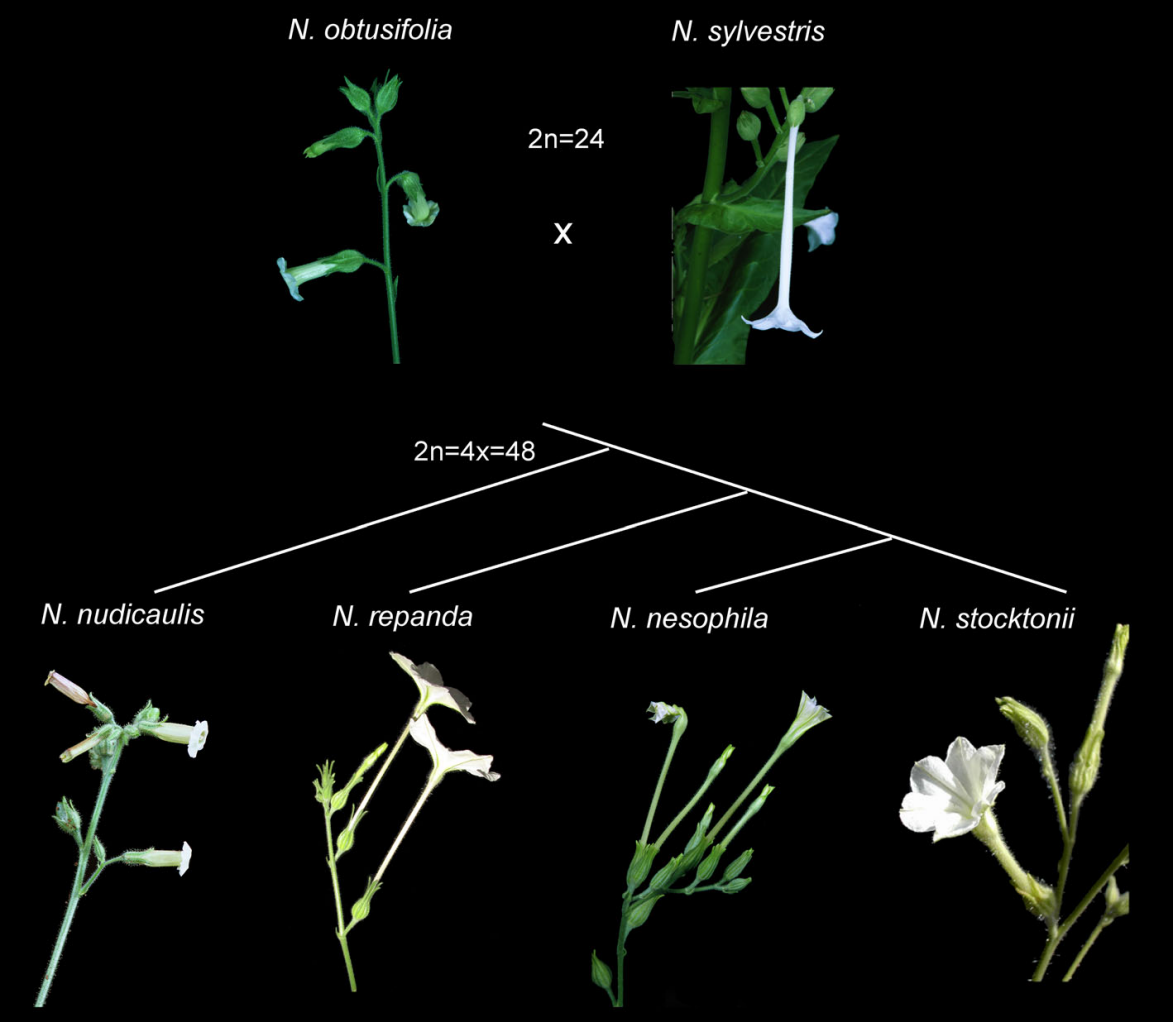
Floral morphology of allotetraploid section *Repandae* species (*Nicotiana nudicaulis*, *N. repanda*, *N. nesophila*, *N. stocktonii*) and the putative progenitor diploid taxa that gave rise to section *Repandae* (*N. obtusifolia*, *N. sylvestris*). Photographs: S. Knapp, E. McCarthy, Y. Lim

**Fig. 2:**
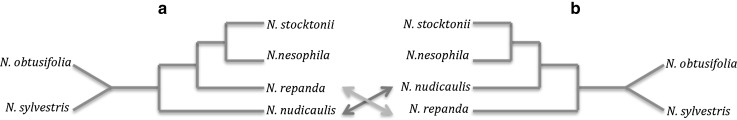
Phylogenetic hypotheses for *Nicotiana* sect. *Repandae* based on **a** nrITS, plastid markers and low-copy nuclear genes (Chase et al. 2003; Clarkson et al. 2004; Kelly et al. 2013; Renny-Byfield et al. 2013) and **b** SSAP profiles of seven repeat families (Parisod et al. 2012)

*Nicotiana* sect. *Repandae* is a group of four species that diverged subsequent to a single allopolyploidisation event, approximately 5 MYA (Knapp et al. 2004; Clarkson et al. 2005). Two species, *N. nesophila* and *N. stocktonii*, are isolated off the coast of Mexico on the Revillagigedo Islands. These are arguably most closely related to the mainland *N. repanda* with which they share similar floral morphology (long floral tubes reminiscent of *N. sylvestris*, the closest extant relative to the maternal progenitor of the section; Fig. 1) and similar genome sizes of around 1C = 5 pg. *Nicotiana nudicaulis* is the final member of the section and has divergent floral morphology with short wide corolla tubes that are much closer to that of *N. obtusifolia* (the closest extant relative of the paternal progenitor; Fig. 1) and a much smaller genome size around 3.5 pg (Leitch et al. 2008). Molecular phylogenetic analyses have consistently reconstructed relationships in the section as (*N. nudicaulis* (*N. repanda* (*N. nesophila* + *N. stocktonii*))), based on the nuclear ribosomal internal transcribed spacer (ITS), plastid markers (coding and non-coding) and low-copy nuclear genes (Chase et al. 2003; Clarkson et al. 2004, 2010; Kelly et al. 2013; Fig. 2). However, a recent analysis of seven transposable element (TE) families using sequence-specific amplified polymorphisms (SSAP) suggested a closer relationship between the island taxa and *N. nudicaulis* (Parisod et al. 2012; Fig. 2), i.e. (*N. repanda* (*N. nudicaulis* (*N. nesophila* + *N. stocktonii*))). Previous analyses of the repetitive portion of the genome of *Nicotiana repanda* and *N. nudicaulis* showed how different elements, with different copy numbers, led to the relative increase (in *N. repanda*) and decrease (in *N. nudicaulis*) of genome size in these species (Renny-Byfield et al. 2013). Additionally, the increase in genome size found in *N. repanda* was due mostly to the accumulation of Ty-3 Gypsy retroelements, which may have had an adverse effect on the ability of genomic in situ hybridisation (GISH) to adequately detect the parental subgenomes as reported previously (Clarkson et al. 2005; Lim et al. 2007; Renny-Byfield et al. 2013). Here we test whether the phylogenetic signal found by Parisod et al. (2012) is characteristic of the repetitive portion of the genome and whether the repeats in the genomes of these allotetraploids are diverging in a way that is distinct from other (i.e. coding) regions of the genome. We investigate all species of the section using high-throughput sequencing (HTS) and a graph-based approach to cluster sequence reads (Novák et al. 2010, 2013), adding HTS data for the Revillagigedo Island taxa, *N. nesophila* and *N. stocktonii*. We also use a refined GISH methodology (Jang and Weiss-Schneeweiss 2015) developed to discriminate highly similar genomes in hybrids/allopolyploids to investigate genome evolution post-polyploidisation in section *Repandae*. This builds on previous work focussing on the substantial differences between *N. repanda* (genome upsizing) and *N. nudicaulis* (genome downsizing) (Renny-Byfield et al. 2013) and the use of repeat clusters as phylogenetic characters (Dodsworth et al. 2015a, b).

## Materials and methods

### Plant material, sequencing and data acquisition

*Nicotiana nesophila* accession 974750097 (from the Botanical and Experimental Garden, Radboud, University of Nijmegen, the Netherlands) and *N. stocktonii* accession TW126 (from the United States Department of Agriculture, North Carolina State University, NC) were used in this study. Genomic DNA was extracted from approximately 100 mg of fresh leaf tissue using a modified CTAB protocol as described in Wang et al. (2013). Libraries were prepared using the TruSeq DNA PCR-Free Library Preparation Kit (version B) and sequenced on a MiSeq at the Genome Centre, Queen Mary University of London with V2 chemistry (300 cycles, 151-bp paired-end reads). New read data (for *N. nesophila* and *N. stocktonii*) have been deposited in the NCBI short-read archive (SRA) as follows: SRP082467. Data for other species were downloaded from SRA as follows: the two remaining species of section *Repandae*—*N. repanda* [GenBank: SRR453021] and *N. nudicaulis* [GenBank: SRR452996], plus the extant relatives of parental lineages of section *Repandae*—*N. sylvestris* [GenBank: SRR343066] and *N. obtusifolia* [GenBank: SRR452993], resulting from Renny-Byfield et al. (2011, 2012, 2013).

### Graph-based clustering using repeat explorer

Sequence reads were screened using a threshold of quality score 20 over 95% of the read length. Newly generated reads for *N. nesophila* and *N. stocktonii* (151 bp) were then trimmed to 95 bp in order to match the read length of previous datasets (Renny-Byfield et al. 2013) that were downloaded from the SRA. Each set of reads was down-sampled to represent 1% of each genome (i.e. coverage of 0.01×) based on flow cytometry 1C values (www.data.kew.org/cvalues): 277,472 reads for *N. sylvestris*, 159,052 reads for *N. obtusifolia*, 366,000 for *N. nudicaulis*, 560,000 for *N. repanda*, 514,526 for *N. stocktonii* and 518,106 for *N. nesophila*. Samples were prefixed with a four-letter code unique to that species and combined to produce a dataset of 2,395,156 reads as input to RepeatExplorer for graph-based clustering (www.repeatexplorer.org; Novák et al. 2013) using the default settings of 90% similarity over 55% of the read length. For full details of the clustering process, see Novák et al. (2010, 2013). The *N. tabacum* plastid genome [GenBank: Z00044.2] was used as a custom database to identify plastid clusters for removal.

### Phylogenetic analysis

A phylogenetic tree was reconstructed from the top 1000 most abundant clusters (repeats) using continuous character parsimony in TNT as described fully in Dodsworth et al. (2015a, b) with the exception that abundances were cube-root-transformed prior to inference. This was in order to make the cluster abundances in the range of 0–65, which is required for input to TNT.

### Deviation from expectation in polyploids

In a neo-allotetraploid, we assume that inheritance is additive (i.e. the allotetraploid genome is the sum of the parental genomes). Thus, the expectation is that repeats in the allotetraploid genome are inherited faithfully in the same abundance from the parental taxa. For *Nicotiana* sect. *Repandae*, the closest extant relatives of progenitor species are *N. sylvestris* (maternal) and *N. obtusifolia* (paternal). The expected cluster size in each of the section *Repandae* polyploids is therefore the sum of the cluster size in *N. sylvestris* and *N. obtusifolia*. The cumulative deviation from expectation was calculated and plotted in log scale using R version 3.0.0 (R Core Team 2013), from smallest (left) to largest cluster size (right), in a similar manner to Renny-Byfield et al. (2013).

### Genomic in situ hybridisation

For cytological investigations, actively growing root meristems of *Nicotiana* sect. *Repandae* species were pre-treated with 0.002 M solution of 8-hydroxyquinoline for 2.5 h at room temperature and 2.5 h at 4 °C, fixed in ethanol-to-acetic acid (3:1) and stored at −20 °C until use. Genomic in situ hybridisation was performed using genomic DNAs of previously identified closest extant relatives of parental diploid species (*N. sylvestris* and *N. obtusifolia*; both 2*n* = 24) as probes (Clarkson et al. 2005; Lim et al. 2007; Renny-Byfield et al. 2013). Genomic DNAs of diploid species were isolated using the CTAB method (Jang and Weiss-Schneeweiss 2015), sheared at 98 °C for 5 min and labelled using either digoxigenin or biotin nick translation kit (Roche). Hybridisation and detection of the probes followed the method of Jang and Weiss-Schneeweiss (2015). Preparations were analysed with an AxioImager M2 epifluorescent microscope (Carl Zeiss, Vienna, Austria), images acquired with a CCD camera and processed using AxioVision version 4.8 (Carl Zeiss, Vienna, Austria) with only those functions that apply equally to whole image. At least 30 well-spread metaphases and prometaphases were analysed for each individual. Cut-out karyotypes are given in Online Resource 1.

## Results and discussion

### Phylogenetic relationships in section *Repandae*

**Fig. 3:**
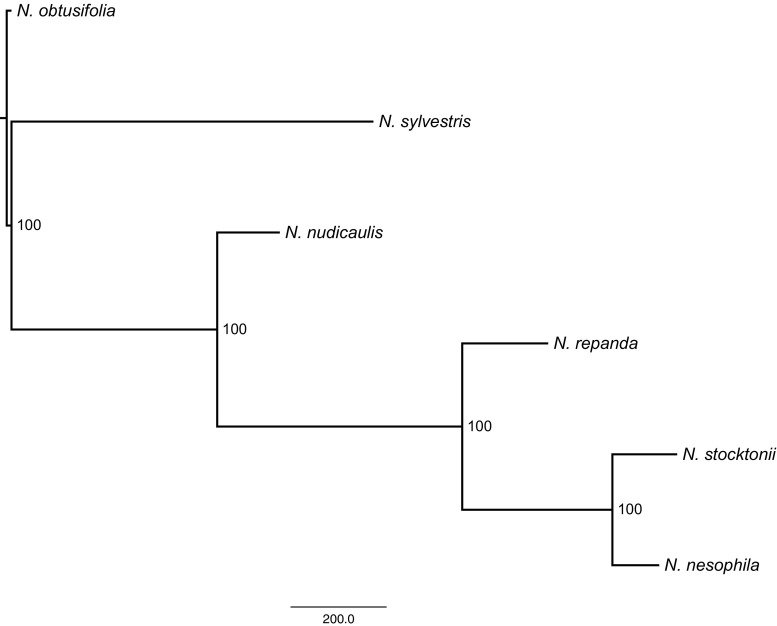
Phylogenetic relationships in *Nicotiana* sect. *Repandae* based on the 1000 most abundant repeat clusters. The single most parsimonious tree is shown, with rooting on *N. obtusifolia* (outgroup). Bootstrap values are shown for each node (10,000 symmetric bootstrap replicates). Branch lengths are proportional to numerical step changes in cluster abundance (*scale bar*)

In contrast to the results of Parisod et al. (2012), we found the same relationship using genome-wide repeat characters as has previously been found with more standard sequence markers and phylogenetic analyses (Fig. 3). This result probably stems from the difference between analysing seven specific repeats versus our analysis of hundreds of different repeat families that are scattered across the genome. One would expect that certain repeats have been subject to selection and/or recent activity (both amplification and deletion) and therefore would not reflect neutral processes, nor would they therefore be indicative of evolutionary history of the taxa. Some such repetitive elements must be within our dataset; however, the relative deviation provided by such repeats is clearly swamped out by the overall signal that reflects evolutionary relationships. The two Revillagigedo Island species, *N. nesophila* and *N. stocktonii*, are the most similar species in terms of genome size, repeat composition and repeat dynamics. This is to be expected as they are thought to have diverged less than 0.2 MYA (Clarkson et al. 2005).

### Patterns of overall repeat divergence reflect evolutionary history

**Fig. 4:**
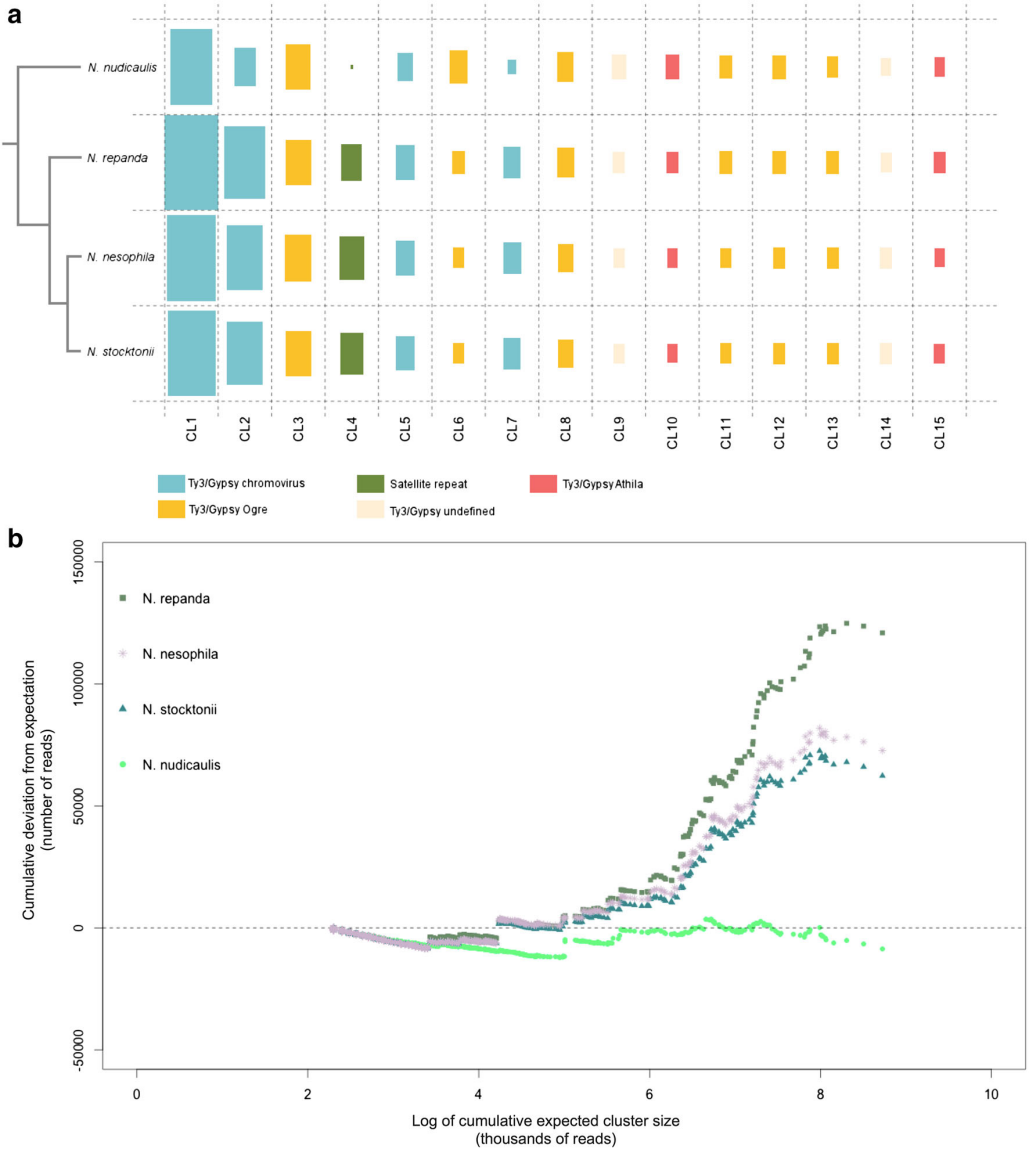
**a** Species-specific proportion of the 15 most abundant clusters in *Nicotiana* sect. *Repandae*. Rectangles are *coloured* according to the type of repetitive element; size of rectangles represents relative genome proportion (largest abundance for a particular taxon fills the entire rectangle, other taxa and clusters are relative proportions of this). Phylogenetic relationships are summarised. **b** Repeat dynamics in *N.* sect. *Repandae*. *Curves* for each species depict the cumulative deviation from expected cluster size, ranked from the smallest (*left*) to largest (*right*) cluster sizes found in each species, presented on a log scale

The similar genome sizes of the island taxa and *N. repanda* reflect similar amplification processes of abundant Ty-3 Gypsy elements, particularly chromovirus-like elements, mostly likely in the most recent common ancestor (MRCA) of these 3 species (Fig. 4a; CL2). There is also a large satellite repeat that represents 2.46-3.25% of the genome (estimated via RE analysis) in the island taxa and *N. repanda*, versus only 0.04% in *N. nudicaulis* in which it is virtually absent (Fig. 4a; CL4). CL4 can therefore essentially be considered a molecular synapomorphy of the (*N. repanda* (*N. stocktonii* + *N. nesophila*)) clade (Fig. 3). This repeat is a satellite of the NNES (HRS60) family previously identified in *Nicotiana nesophila* by Koukalova et al. (2010), with the same distribution amongst *Repandae* species, similar genomic abundance and subtelomeric localisation. NNES originated by amplification of a low-copy repeat present in the maternal progenitor *N. sylvestris*—NSYL1 (Koukalova et al. 2010), and using phylogenetic analysis of the most abundant CL4 monomer the CL4 satellite could be confirmed as NNES (Online Resource 2). The ability of repeat abundances to recapitulate evolutionary history when analysed as phylogenetic characters, e.g. Dodsworth et al. (2015a), suggests that broad patterns of repeat dynamics (i.e. accumulation, deletion, turnover) would also reflect phylogenetic distance. Here we show that more closely related species of *Nicotiana* allotetraploids do indeed show similar repeat dynamics in general (Fig. 4). In the context of allotetraploid evolution post-polyploidisation, the three species that show genome size increases (*N. repanda*, *N. nesophila*, *N. stocktonii*) have a similar shape to their profiles of repeat divergence across the expected cluster size (Fig. 4), whereas the genome downsizing *N. nudicaulis* shows a very different profile. Parts of these profiles are the same for all taxa, especially in the deviation from expected cluster size for small clusters (initial part of the graph), which may reflect some general trends towards the loss of DNA from low abundance repeats (Fig. 4b). This perhaps reflects diploidisation processes associated with loss of sequences subsequent to polyploidy (Wendel 2015), sometimes reported as preferential deletion from one of the parental subgenomes, of both genes (known as biased fractionation; Freeling et al. 2012) and repeats (Renny-Byfield et al. 2015).

**Fig. 5:**
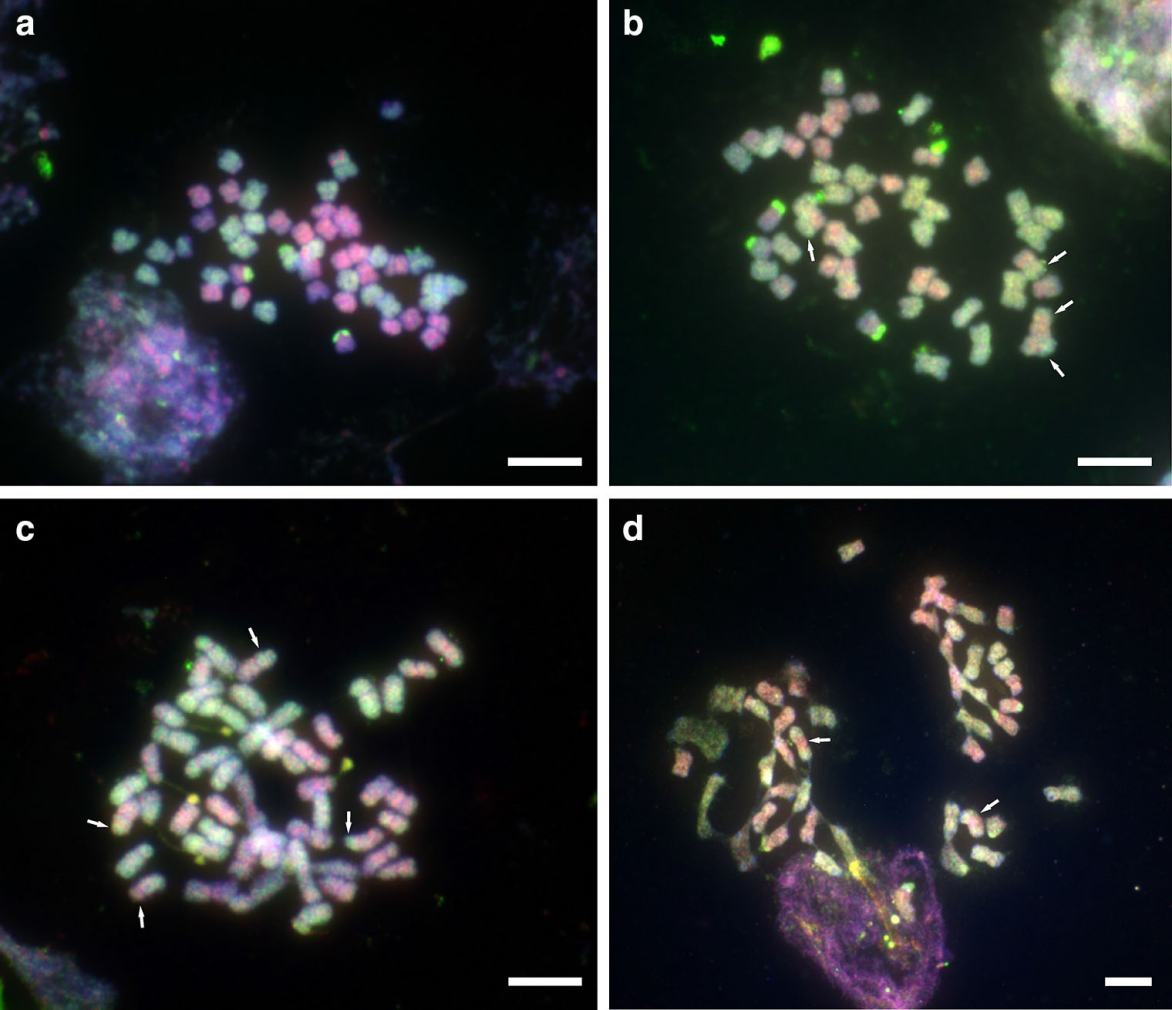
Genomic in situ hybridisation in the four species of *Nicotiana* sect. *Repandae*, probed with the parental gDNAs of *N. sylvestris* (*green*) and *N. obtusifolia* (*red*). **a** *N. nudicaulis*. **b** *N. stocktonii*. **c** *N. nesophila*. **d** *N. repanda* (incomplete plate with two missing chromosomes). *Arrows* indicate exchanges between parental subgenomes. *Scale bar* 5 μm

Variance in repeat abundance from expectation at the larger end of cluster size reflects the differential amplification of particular repeats, with the increase in *N. repanda*, *N. nesophila* and *N. stocktonii* due mostly to the increase of various Ty-3 Gypsy retroelements, and in particular chromoviruses (Fig. 4). These retroelements are distributed throughout the genome, and this is the likely reason why GISH performs less adequately in these species when compared to *N. nudicaulis* (Fig. 5; Online Resource 1; Renny-Byfield et al. 2013), though using a new methodology (Jang and Weiss-Schneeweiss 2015) it is clearly possible to distinguish parental genomes in the other 3 species as well, and in some cases even see clear translocations (Fig. 5). Translocations were occasionally seen in all species except for *N. nudicaulis*. In *N. nudicaulis*, the two parental subgenomes can be more easily distinguished (*N. sylvestris* and *N. obtusifolia*), due to a lack of highly abundant dispersed retroelements (Ty-3 Gypsy) in this species when compared to the other 3 species. These elements (i.e. CL1), which are not found in either parental lineage in high abundance, therefore reflect the process of genome turnover (Lim et al. 2007; Renny-Byfield et al. 2013) and homogenisation that distinguishes the genome from that of a newly formed allopolyploid. Such processes may be considered to be part of the cycle (Wendel 2015) that returns the allopolyploid genome to a more diploid-like state. All species of *N.* sect. *Repandae* show relatively few translocations following allopolyploidy, especially so after 5 million years and when compared to more recent polyploids formed only tens or thousands of years ago in *Tragopogon* and *Brassica* (Gaeta et al. 2007; Chester et al. 2012).

## Conclusions

Phylogenetic analysis of the abundance of different repeats in the genomes confirms relationships in *Nicotiana* sect. *Repandae* as (*N. nudicaulis* (*N. repanda* (*N. stocktonii* + *N. nesophila*))). This result reflects much previous work using standard phylogenetic markers, summarised in Renny-Byfield et al. (2013). Additionally, the patterns of repeat dynamics in section *Repandae* species clearly reflect phylogenetic distance of these species from one another. This is an extension of the phylogenetic signal in abundance of repeat types (e.g. Piednoël et al. 2012, 2013; Novák et al. 2014; Macas et al. 2015) and shows that the overall patterns of element accumulation/deletion/stasis are broadly similar in closely related taxa. For example, the profiles for *N. nesophila* and *N. stocktonii*, the most recently diverged taxa, are almost identical, and together they most closely resemble *N. repanda*, to which they are sister (Fig. 4). These three taxa are very divergent from *Nicotiana nudicaulis*, in repeat profile, as well genome size, with *N. nudicaulis* having a smaller and less restructured genome, being sister to the other three species. Previous SSAP patterns (Parisod et al. 2012) therefore reflected the novel activity of a very limited number of retroelement families, the dynamics of which are not directly indicative of shared evolutionary history.

## Information on Electronic Supplementary Material

**Online Resource 1.** Cut-out karyotypes of the four allotetraploid species of *Nicotiana* sect. *Repandae* based on GISH and phase contrast images. Chromosomes were probed with the parental gDNAs of *N. sylvestris* (green) and *N. obtusifolia* (red). **a** *N. nudicaulis*. **b** *N. stocktonii*. **c** *N. nesophila*. **d** *N. repanda* (incomplete plate; two missing chromosomes are indicated by stars). Arrows indicate small intergenomic exchanges. All images (left) are identical to those shown in Fig. 4. Scale bar, 5 μm.

**Online Resource 2.** Phylogenetic analysis of the most abundant CL4 monomer sequence, amongst members of the HRS60 family previously identified in *Nicotiana* sect. *Repandae* (Koukalova et al. 2010). Maximum likelihood tree with 100 bootstrap replicates, conducted in PhyML/Seaview with GTR model and 4 rate classes.

### Electronic supplementary material

Below is the link to the electronic supplementary material.

Supplementary Figure S1 Cut-out karyotypes of the four allotetraploid species of *Nicotiana* sect. *Repandae* based on GISH and phase contrast images. Chromosomes were probed with the parental gDNAs of *N. sylvestris* (green) and *N. obtusifolia* (red). (A) *N. nudicaulis*. (B) *N. stocktonii*. (C) *N. nesophila*. (D) *N. repanda* (incomplete plate; two missing chromosomes are indicated by stars). Arrows indicate small intergenomic exchanges. All images (left) are identical to those shown in Figure 4. Scale bar, 5 μm (PDF 1346 kb)

Supplementary Figure S2 Phylogenetic analysis of the most abundant CL4 monomer sequence, amongst members of the HRS60 family previously identified in *Nicotiana* sect. *Repandae* (Koukalova et al. 2010). Maximum likelihood tree with 100 bootstrap replicates, conducted in PhyML/Seaview with GTR model and 4 rate classes (PDF 94 kb)
