# Supplementary figures and images for: Genome-wide repeat dynamics reflect phylogenetic distance in closely related allotetraploid Nicotiana (Solanaceae)
Source: Plant Syst Evol. 2016 Nov 1;303(8):1013–20. doi: 10.1007/s00606-016-1356-9 (PMC6961477; doi:10.1007/s00606-016-1356-9)

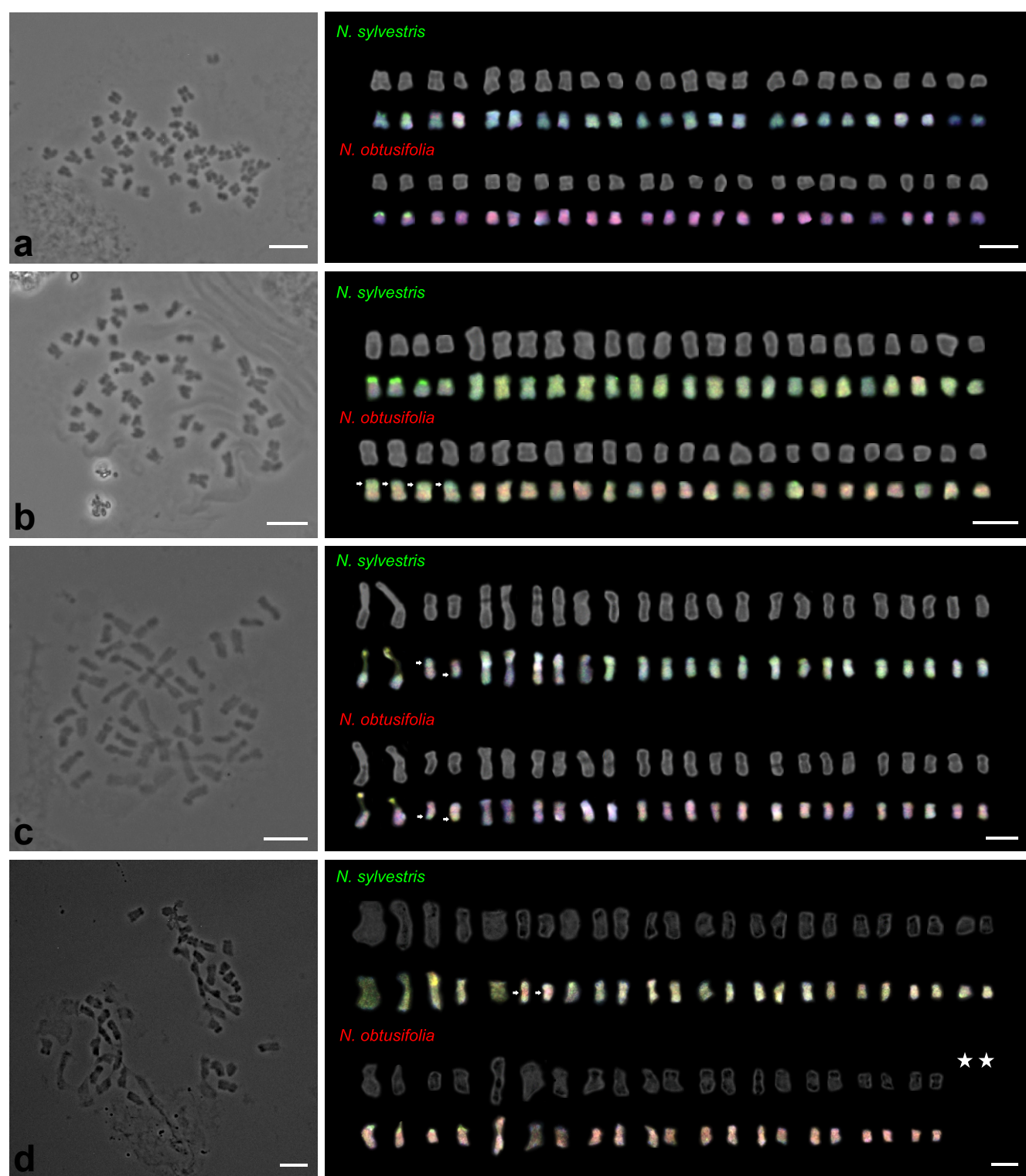

Supplement: Supplementary file 1 — Cut-out karyotypes of the four allotetraploid species of Nicotiana sect. Repandae based on GISH and phase contrast images. Chromosomes were probed with the parental gDNAs of N. sylvestris (green) and N. obtusifolia (red). (A) N. nudicaulis. (B) N. stocktonii. (C) N. nesophila. (D) N. repanda (incomplete plate; two missing chromosomes are indicated by stars). Arrows indicate small intergenomic exchanges. All images (left) are identical to those shown in Figure 4. Scale bar, 5 μm (PDF 1346 kb) [file 606_2016_1356_MOESM1_ESM.pdf]
