# Supplementary material for: Genome-wide repeat dynamics reflect phylogenetic distance in closely related allotetraploid Nicotiana (Solanaceae)
Source: Plant Syst Evol. 2016 Nov 1;303(8):1013–20. doi: 10.1007/s00606-016-1356-9 (PMC6961477; doi:10.1007/s00606-016-1356-9)
Supplement: Supplementary file 2 — Phylogenetic analysis of the most abundant CL4 monomer sequence, amongst members of the HRS60 family previously identified in Nicotiana sect. Repandae (Koukalova et al. 2010). Maximum likelihood tree with 100 bootstrap replicates, conducted in PhyML/Seaview with GTR model and 4 rate classes (PDF 94 kb) [file 606_2016_1356_MOESM2_ESM.pdf]

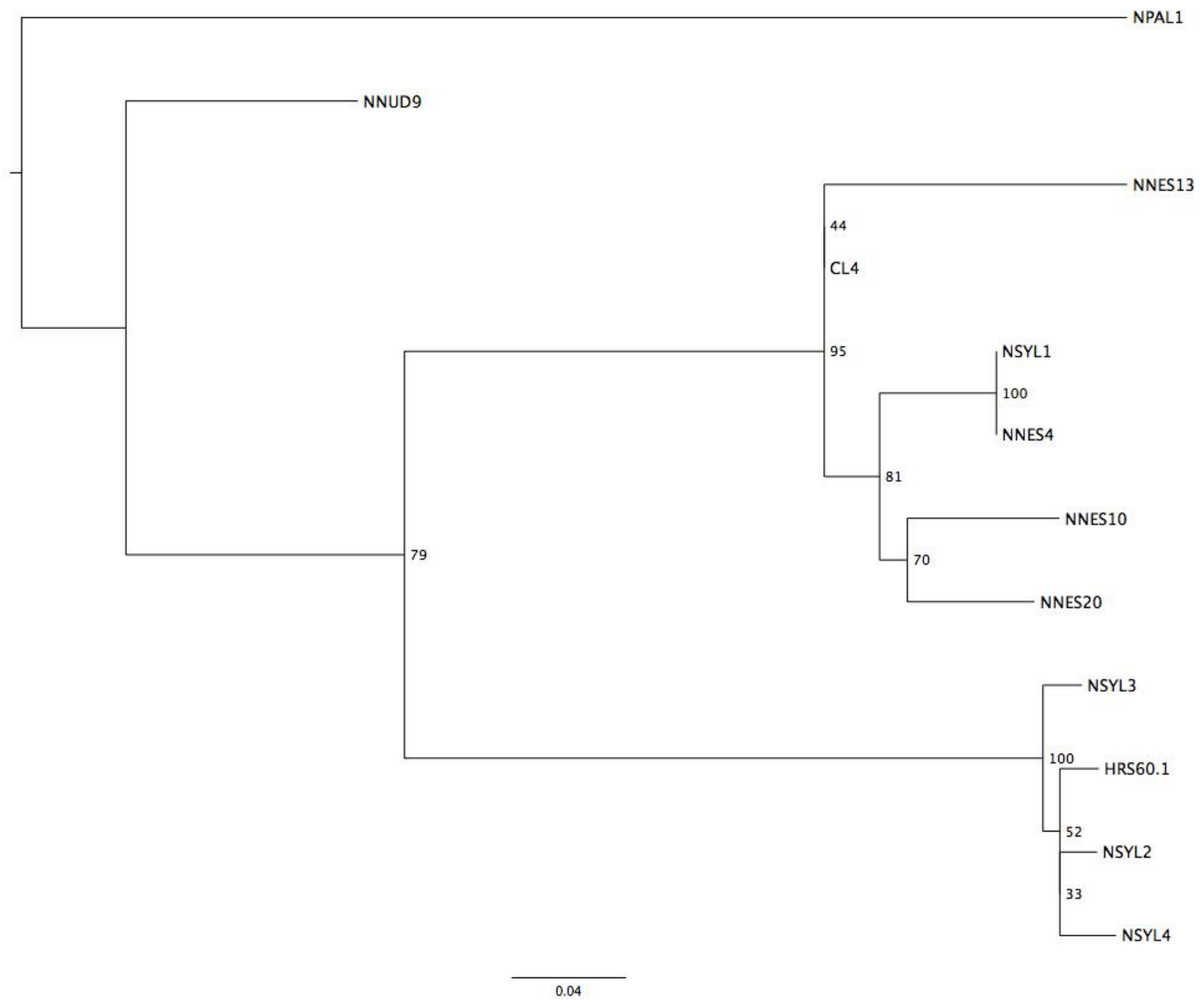

Online Resource S2. Phylogenetic analysis of CL4 satellite repeat monomer, within the context of the HRS60 family satellites from *Nicotiana*, using representative sequences from Koukalova *et al*. (2010). Maximum likelihood tree conducted using PhyML in Seaview, with 100 bootstrap replicates, GTR model with 4 rate classes, indels and gaps coded as missing data. Tree shows that the most abundant CL4 sequence is clearly part of the NNES subtelomeric satellite repeat family, as previously identified by Koukalova *et al*. (2010).

Further details:

Reconstructed monomer sequence for CL4:
>CL4Contig640
CTTTGAAACTAAAAATCCGCACGAGTCCCAAATTTTCGTGTACTATAGCTCACGCCTTCG
GCTGTGGGCCCGGGACCCCGGACCCGGATCGCCTAATATTTTACCGGGACCTCGCATACT
AACTAAGGAACCATTTTCACCAAACCGCCGTGCTCAAATAATAATATTTTGCATTTTACG
GCCATTAAAACTCAAAATCCGCGCGAGTCCCAAATTTTCGTGTACTATAGCTCACGCCTT
CGGCCGTGGGCCCGGGACCCCGGACCCGGATCGCCTAATATTTTACCGGGACCTCGCATA
CTGCCTAAGGAACCATTTTCACCAAACCTCCATGCTCAAATATTAATATTTTGCATTTTA
CGGCCATTAAAACTCAAAATCCGCGCGAGTCCCAAATTTTCGTGTACTATAGCTCACGCC
TTCGGCCGTGGGCCCGGGACCCCGGACCCGGATCGCCTAATATTTTTCCGGGACCTCGCA
TACTACCTAAGGAACCATTTTCACCACACCTCCATGCTCAAATATTATTTTTCGCATTTT
ACGGTCATCAAAACTC

- The most abundant contig (Contig 640) was taken from the CL4 cluster directory in the output of RepeatExplorer and the contig file sorted by genome representation. This contig had an average coverage of 5067.3, an order of magnitude more than the next contig (~700), therefore likely representing the most abundant sequences within the CL4 cluster.
- Using a dotplot in Geneious, plus the software Tandem Repeats Finder (https://tandem.bu.edu/trf/trf.html) , the most abundant monomer sequence found within the most abundant contig was reconstructed as above.
- Previous sequences from Koukalova *et al*. (2010) were downloaded from GenBank with the following accession numbers: DQ536094.3 – NPAL1; FJ664193.1 – NNUD9; FJ664187.1 – NNES13; FJ664189.1 – NSYL1; DQ536093.1 – NNES4; FJ664186.1 – NNES10; FJ664188.1 – NNES20; FJ664192.1 – NSYL3; X12489.1 – HRS60.1; FJ664190.1 – NSYL2; FJ664191.1 – NSYL4.
- These sequences were aligned using MAFFT online version (http://mafft.cbrc.jp/alignment/server/index.html) and checked in Seaview prior to phylogenetic analysis as described above. The final tree was saved and edited briefly in FigTree for visualisation purposes.
